# Inhibited Lipophagy Suppresses Lipid Metabolism in Zebrafish Liver Cells

**DOI:** 10.3389/fphys.2019.01077

**Published:** 2019-08-21

**Authors:** Jing Wang, Si-Lan Han, Dong-Liang Lu, Ling-Yu Li, Samwel Mchele Limbu, Dong-Liang Li, Mei-Ling Zhang, Zhen-Yu Du

**Affiliations:** ^1^Laboratory of Aquaculture Nutrition and Environmental Health, School of Life Sciences, East China Normal University, Shanghai, China; ^2^Department of Aquatic Sciences and Fisheries Technology, University of Dar es Salaam, Dar es Salaam, Tanzania

**Keywords:** zebrafish liver cells, lipophagy, lipid metabolism, fatty acid β-oxidation, esterification

## Abstract

Lipophagy degrades lipid droplets (LDs) through the lysosomal degradative pathway, thus plays important roles in regulating lipid metabolism in mammals. However, information on the existence and functions of lipophagy in fish lipid metabolism is still limited. In the present study, we confirmed the existence of lipophagy by observing the structures of LDs sequestered in autophagic vacuoles in the zebrafish liver cell line (ZFL) via electronic microscopy. Moreover, starved cells increased the mRNA expression of the microtubule-associated protein 1A/1B light chain 3 beta (*LC3*), which is a marker protein for autophagy and protein conversion from LC3-I to LC3-II. Inhibiting autophagy with chloroquine increased significantly the LDs content and decreased fatty acid β-oxidation and esterification activities in the ZFL cells cultured in the fed state. Furthermore, inhibiting autophagy function downregulated the mRNA expression of the genes and their proteins related to lipid metabolism. Altogether, the present study verified the existence of lipophagy and its essential regulatory roles in lipid metabolism in fish cells.

## Introduction

Lipophagy is a cellular process, which delivers cytoplasmic lipid droplets (LDs) to the lysosome, leading to their degradation (Singh et al., 2009). Autophagy-related genes (*ATG*) are required for this dynamic process to control a series of steps consisting of initiation, vacuole membrane nucleation and elongation, and autophagosome formation (Jin and Klionsky, 2013). In mammals, lipophagy has been well demonstrated as an important cellular process involved in lipid metabolism and energy homeostasis. For example, lipophagic activity was upregulated dramatically after starvation in mice in order to reduce LDs and supply more energy (Singh et al., 2009; Weidberg et al., 2009). Conversely, lipid content increased when lipophagy was blocked either genetically (Du et al., 2001; Komatsu et al., 2005) or genetically and pharmacologically (Singh et al., 2009). Loss of autophagic function has been correlated with some lipid dyslipidaemia syndromes, such as fatty liver and obesity (Yang et al., 2010; Czaja et al., 2013; González-Rodríguez et al., 2014). Correspondingly, enhancing autophagy activity by using pharmacological approaches has been reported to protect against chronic ethanol-induced hepatotoxicity and steatosis (Ding et al., 2010; Lu and Cederbaum, 2015) and prevent non-alcoholic fatty liver in cafeteria diet-induced rats (Parafati et al., 2015). Therefore, lipophagy is a promising therapeutic target for treating metabolic disorders. However, the detailed roles of autophagy in lipid metabolism such as lipid catabolism and esterification have not been well addressed in mammals and fish.

Many farmed fish and mammals are currently affected by metabolic disorders characterized by severe fatty livers (Du et al., 2001, 2010). Metabolic disorders cause several adverse effects, such as lowered growth rate and stress resistance in fish (Morais et al., 2001; Du et al., 2006). Therefore, approaches to reduce lipid accumulation in fish liver have attracted the attention of many fish physiologists. Lipophagy dysfunction has been verified to participate in the progress of steatosis in mammals (Ding et al., 2010; Lu and Cederbaum, 2015). However, information on the existence and functions of lipophagy in lipid metabolism in fish liver is currently limited. For the first time, we verified the existence of lipophagy in zebrafish liver in our recent work (Wang et al., 2018). In the present study, we used zebrafish liver cell line (ZFL) to address how lipophagy is regulated under different experimental conditions in fed and starved cells, while providing more experimental detail with respect to our previous study.

Therefore, in the present study the existence of lipophagy in ZFL cells was verified by using specific lipophagy-related cellular structures through electron microscopy (EM) and the expression of LC3, which is a marker protein succesfully used to assay autophagic activity both in zebrafish and in mammals (He et al., 2009; Yabu et al., 2012). We further used chloroquine (CQ), a widely biochemical inhibitor of lysosomal activity in lipophagy (Sinha et al., 2012; Skop et al., 2012), to block autophagy and lipophagy. Finally, we assessed the lipid metabolism-related biochemical reactions and genes expression under varying lipophagic activities in order to understand the roles and functions of lipophagy in lipid metabolism. Through these assays, we verified the existence of lipophagy in ZFL cells and its critical functions in lipid metabolism. These findings enlighten our knowledge on the development of metabolic diseases such as fatty liver in fish.

## Materials and Methods

### Animal Ethics

We followed all applicable institutional and/or national guidelines for the care and use of animals. Thus, the experiments used in this study were approved by the Committee on the Ethics of Animal Experiments of East China Normal University and followed the Guidance for the Care and Use of Laboratory Animals formulated by the Ministry of Science and Technology in China.

### ZFL Cell Line Culture

Zebrafish liver cell line cell line was kindly provided by Professor Zhi-Gang Zhou (Chinese Academy of Agricultural Sciences, China). An L15-DMEM-F12 (LDF) medium consisting of 50% Leibovitz’s medium (L-15, Gibco, United States), 35% Dulbecco’ Modified Eagle medium (DMEM, Gibco, United States), and 15% Faighn’s Modification nutrient mixture (F-12, Gibco, United States) was used to prepare the medium for cell culture. Cells were cultured in LDF complete medium containing 6% fetal bovine serum (FBS, Biological Industries), 15 mM HEPES buffer solution (Gibco, United States) and 50 Unit/mL penicillin and 50 μg/mL streptomycin (Gibco, United States) at 28°C in 5% CO_2_. Cells (∼1 × 10^5^ cells per cm^2^) were plated in 6-well plates. When the cells were attached, the culture medium was replaced with the LDF complete medium containing 250 μM oleic acid [OA, Sigma, United States, containing a 1.5:1 molar ratio of free fatty acids (FFAs) to bovine serum albumin (BSA)] for 24 h in order to allow high LDs accumulation. In the experiments to verify the existence of lipophagy in cells, OA-treated cells were cultured in the DMEM without glucose and serum and/or co-treated with CQ (Sellect, United States, 50 μM) for 24 h. In order to assess the physiological roles of lipophagy in fed state, the cells were cultured in a complete LDF medium and/or co-treated with 50 μM CQ for 24 h. We conducted a preliminary experiment to test the effects of different CQ concentrations (10, 50, 100, 200 μM) on LDs accumulation and cell apoptosis during a 24 h-culture and found that, a 50 μM CQ concentration caused LDs accumulation without impairing significantly cell growth (data not shown). Moreover, the 50 μM CQ concentration has been used in a 48 h-culture study in zebrafish embryo and was deemed safe (Cui et al., 2012). In fact, CQ had been widely used in zebrafish studies with different concentrations up to 100 μM (Feng et al., 2010; Ruparelia et al., 2014; van der Vaart et al., 2014). Therefore, in the present study, a 50 μM CQ concentration was used for the different experiments.

### Visualization and Imaging of LDs Structures

Zebrafish liver cell line cells were fixed with 2.5% glutaraldehyde in 100 mM phosphate buffer (pH 7.4) and post-fixed by using 1% osmium tetroxide. They were dehydrated in a series ascending of alcohol concentrations followed by cutting and staining the sections by using uranylacetate and lead citrate as reported previously (Sinha et al., 2012). Images were obtained by using the JEM1200EX transmission electron microscope (EM).

### Histological Analysis

Zebrafish liver cell line cells were fixed with 4% paraformaldehyde, followed by staining using BODIPY 493/503 (D3922, Invitrogen) for 15 min in a 10 ug/mL concentration at room temperature, as described earlier (Zhou et al., 2014). The images were captured from the stained cells by using the Fluorescence microscopy (Nikon, Japan) and quantified by using the Image J software.

### Isolation of RNA, Synthesis of cDNA, and Quantitative Real-Time PCR

The total RNA of ZFL cells were isolated by using TRIzol reagent (Invitrogen, United States). Immediately, the cDNAs were synthesized from each RNA sample (1 μg) by a PrimeScript RT reagent kit (TaKaRa, Japan). Quantitative real-time polymerase reaction (qRT-PCR) was conducted on a CWBIO UItraSYBR Mixture. The qRT-PCR was performed by using a 20 μL reaction system containing 10 μL 2 × UltraSYBR Mixture, 0.4 μL for forward and reverse primers (10 μM), 2 μL of cDNA sample, and 7.2 μL of RNase-Free water. The PCR reactions were as follows: 95°C for 10 min, followed by 35 cycles consisting of 95°C for 15 s, 60°C for 30 s, and 72°C for 30 s. The resulting melting curves were checked to confirm whether only one fragment was amplified. The values of threshold (C_*T*_) were determined by the CFX Connect Real-Time System (Bio-Rad). All primers for genes used in the present study were synthesized by Sangon Biotech (Shanghai, China) and the details are presented in Supplementary Table S1. Elongation factor 1 alpha (*EF1*α) and β-*actin* were used as the genes for normalization (Tang et al., 2007; Lang et al., 2016). The relative expression of genes were calculated by using the 2^–ΔΔ*CT*^ method, thereof, ΔCt = Ct_*target*_ – (Ct_*EF*__1__α_ + Ct_β__–*actin*_)/2.

### Western Blotting (WB) Analysis

Cell lysates were prepared by using the radio immunoprecipitation assay (RIPA) lysis buffer (Beyotime Biotechnology, China) for western blottting analysis. Proteins were separated by using sodium dodecyl sulphate-polyacrylamide gel electrophoresis (SDS-PAGE). Membranes were incubated by using the following primary antibodies: rabbit anti-LC3 (#4108, CST, United States), rabbit anti-sequestosome 1 (p62/SQSTM1, #5114, CST, United States), rabbit anti-adipose triglyceride lipase (ATGL, #2138, CST, United States), rabbit anti-phospho-hormone sensitive lipase (HSL, D151508, BBI, China) and rabbit anti-peroxisome proliferator-activated receptor (PPAR)-α (AB126285, Abcam, United Kingdom). Autophagy marker (LC3) was recognized by using the rabbit polyclonal anti-LC3A/B. The LC3-I and LC3-II were distinguished owing to their differences in molecular weights (16 and 14 kDa, respectively). Western blot for GAPDH was used as the loading control. The WB images were obtained by using the Odyssey CLx Imager (Licor, United States).

### Immunofluorescence Analysis

Zebrafish liver cell line cells were fixed in 4% paraformaldehyde for 30 min, blocked by using the goat serum for 30 min at 37°C and incubated by using the rabbit polyclonal anti-LC3A/B overnight at 4°C. On the next day, the cells were incubated by using the corresponding anti-rabbit IgG (H + L), F(ab’)_2_ Fragment (Alexa Fluor 594 conjugate) secondary antibody (8890, Cell Signaling Technology, United States). The LDs were stained by using BODIPY 493/503 and the nucleus were dyed by using 4’,6-diamidino-2-phenylindole (DAPI) fluorescent stain. The images were observed by using a confocal microscope Leica SP8 with a 63× objective.

### Fatty Acids β-Oxidation Assay

Fatty acids (FA) β-oxidation activity was measured as described previously by Du et al. (2010). Briefly, cells were co-treated by using 50 uM CQ for 24 h and washed gently twice by using phosphate-buffered saline (PBS) buffer. The reaction was initiated by adding one mL of LDF supplement containing 50 μM [1-^14^C] OA (0.3 × 10^6^ dpm/mL) BSA complex at a 1.5:1 molar ratio, 0.2 mM L-carnitine, 10 μM cytochrome C, and 0.2 mM malic acid. One mL of 10% (w/v) perchloric acid was added to stop the reaction after incubation at 28°C for 2 h. The resulting contents were transferred into tubes in ice and then they were filtered by using a Millipore filter (0.45 μm pore size). The water-soluble products containing ^14^C from FA β-oxidation were assayed for radioactivity in a liquid scintillation spectrometer MicroBeta^2^ Plate Counter (Perkin, United States). The wastes of isotopes were collected and treated by a professional company for proper disposal.

### Fatty Acids Esterification Assay

The FA esterification activities were measured as described previously by Du et al. (2010). Briefly, cells were co-treated by using 50 uM CQ for 24 h and then washed gently with PBS buffer. The reaction was initiated by adding 1 mL of LDF supplement containing 50 μM [1-^14^C] OA (0.3 × 10^6^ dpm/mL)-BSA complex at a 1.5:1 molar ratio and 100 nM insulin. After incubation for 2 h, all the media with cells were transferred into glass tubes and then 3 mL of chloroform/methanol (2/1, v/v) were added to extract the lipids. The lipid classes were separated by thin-layer chromatography (TLC). Firstly, total lipid was dissolved in 10 μl methylene chloride and carefully dripped on the activated silica gel thin-layer (20 cm × 20 cm × 0.2 mm, F254, Merck, German). Polar and neutral ester was separated with (methyl acetate/isopropanol/chloroform/methyl alcohol/0.25% KCl, 25:25:25:10:9, by vol) and (*n*-heptane/diethyl ether/acetic acid, 55:45:1, by vol) in sequence. Afterward, the TLC plate was air dried and put into a glass jar with iodine vapor. After 15 min, the visible lipid bands on the plate were scrapped from the plates for radioactivity measurement. The wastes of isotopes were collected and treated by a professional company for proper disposal.

### Statistical Analyses

All data were obtained from at least three independent experiments. Results are presented as mean ± SEM. (*n* ranges from 3 to 6). Data were tested for normality by using Shapiro–Wilk test and homoscedasticity by using Levene’s test. The Student’s *t*-test was used to test for the significant differences between means of measured parameters (SPSS 19, IBM, Armonk, NY, United States). Differences were considered significant at the level of *p* < 0.05.

## Results and Discussion

### Starvation Induced Lipophagy in ZFL Cells

**FIGURE 1:**
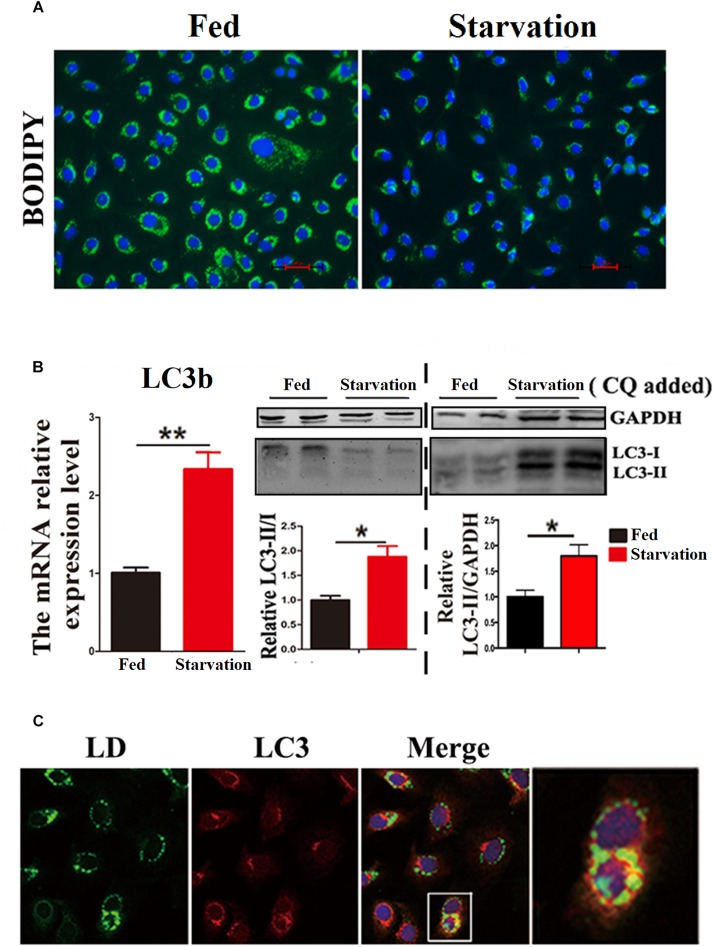
Starvation induces lipophagy in ZFL cell line. **(A)** Lipid droplets (LDs) stained with BODIPY 493/503 (green) in ZFL cell line in starvation condition. **(B)** The mRNA expression, immunoblot and densitometric analysis of LC3 in the samples in ZFL cell line. (^∗^*P* < 0.05, ^∗∗^*P* < 0.01, *n* = 6). Error bars, SEM. **(C)** Co-localization of BODIPY 493/503 (green) with LC3 (red) in ZFL cell line in starvation condition. Nuclei are highlighted with 4, 6-diamidino-2-phenylindole (DAPI).

**FIGURE 2:**
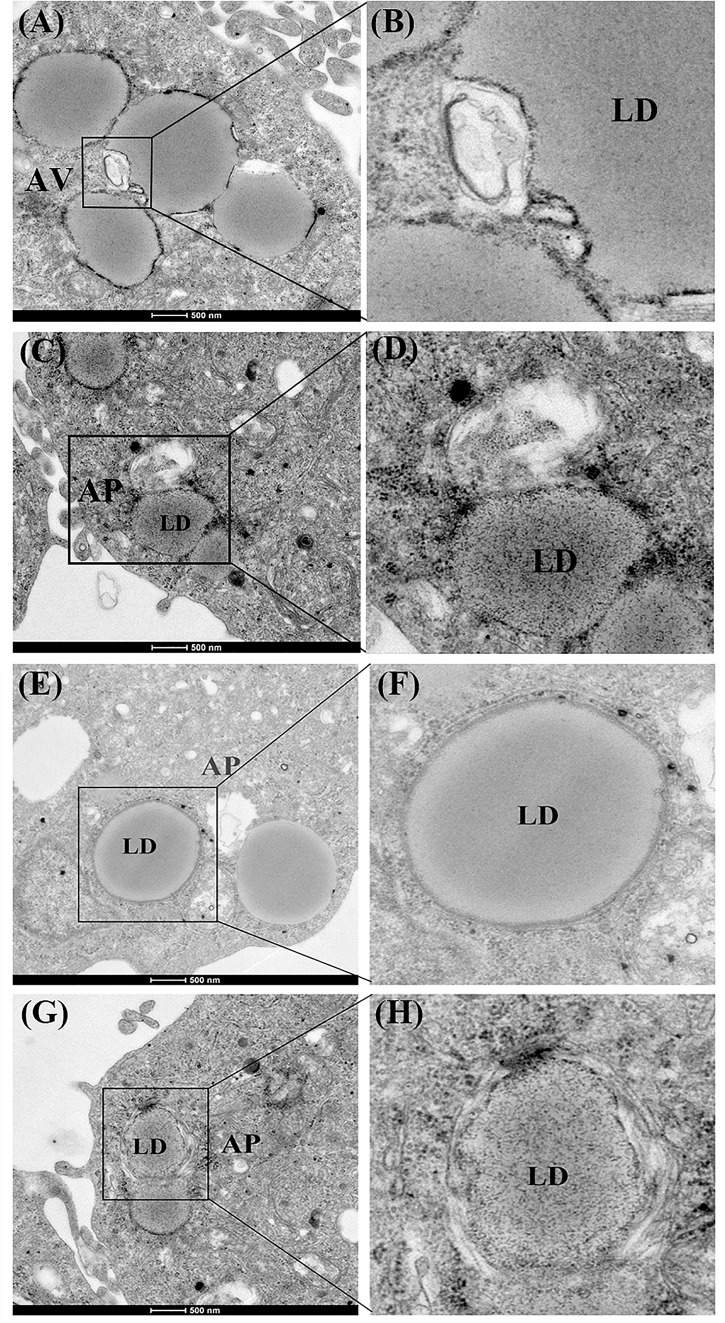
Electron micrographs of ZFL cells. **(A,B)** The structure of an autophagic vesicole (AV). **(C–H)** The structure of an autophagosome containing a lipid droplet (AP). N, nucleus; ER, endoplasmic reticulum; Mit, mitochondria.

First, we investigated whether lipophagy can be induced by starvation in ZFL cells. ZFL cells were pre-incubated by using OA to accumulate LDs. Results indicated that, during starvation the number of LDs stained by BODIPY 493/503 decreased significantly (Figure 1A). We further found that the mRNA expression of *LC3b* and the conversion of the protein from LC3-I to LC3-II increased significantly in starved ZFL cells compared with the fed state (Figure 1B). Like in mammals, zebrafish *LC3* also undergoes similar post-translational modifications. The cytosol form of LC3-I binds covalently to phosphatidyl ethanolamine to form LC3-II and attaches to autophagosome membrane in response to autophagy stimulation (He et al., 2009). To determine precisely the amount of LC3-II, we added CQ as a lysosome inhibitor, in the ZFL cells to prevent the degradation of LC3-II and then we quantified the amount of LC3-II in the fed or starved cells by comparing with GAPDH (Figure 1B). The results indicated that, the amount of LC3-II increased significantly in the starvation state compared to the fed state, confirming again the existence of autophagy in the starved ZFL cells. Furthermore, we showed increased LDs/LC3 co-localization in starved cells by using double immunofluorescence analyses, indicating a direct association between LDs and autophagosomes (Figure 1C). We further used the EM as a common gold standard method in autophagy studies that is used to show LDs sequestration via autophagic vacuoles (Swanlund et al., 2010). We found different subcellular structures associated with lipophagy in the starved ZFL cells, such as the double-membrane structured-autophagic vesicles (AVs) linking with a LD and the autophagosome enclosed a small LD (Figure 2). Taken together, these results confirm that lipophagy exists in ZFL cells and plays significant roles in the LDs dynamic changes, similar to results obtained in our previous study using fasted live zebrafish (Wang et al., 2018). These results suggest that lipophagy characteristics can be determined both in fasted fish and cells. It should be noted that, beside mammals, lipophagy has also been reported during spermatogenesis in the Chinese soft-shelled turtle (Ahmed et al., 2016). Therefore, it is reasonable to suggest that lipophagy might be an evolutionarily conserved basic cellular process in vertebrates.

### Inhibited Lipophagy Caused LDs Accumulation and Decreased Lipid Metabolism Biochemical Activities in ZFL Cells

**FIGURE 3:**
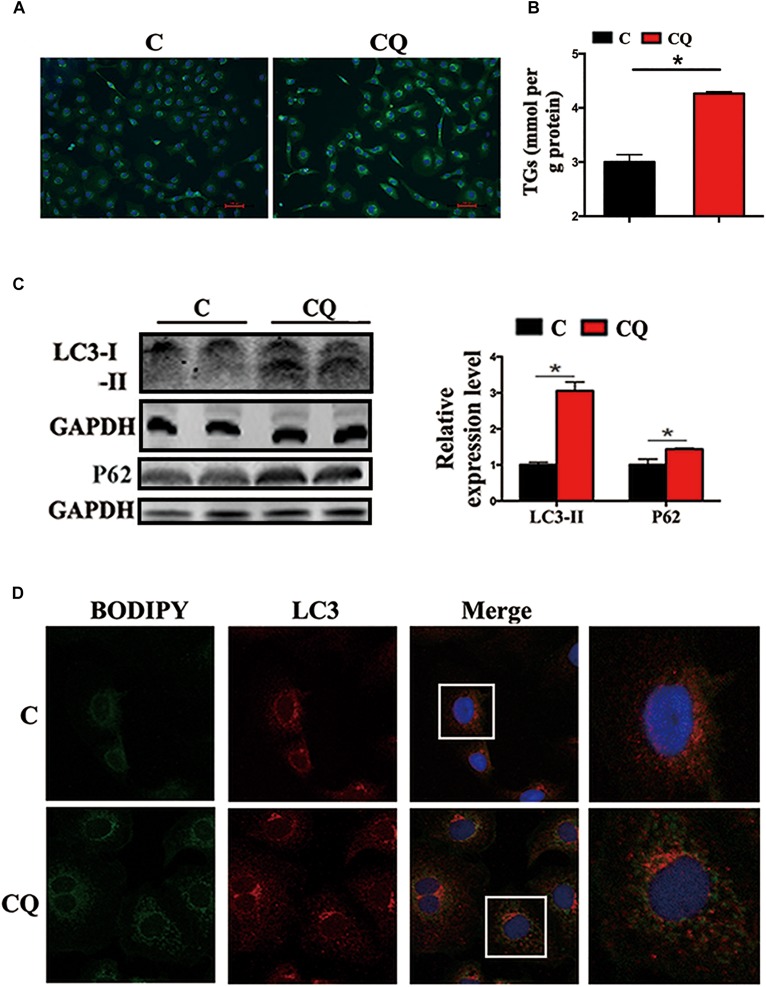
Inhibited lipophagy causes accumulation of lipid droplets in ZFL cell line. **(A,B)** Lipid droplets (LDs) stained with BODIPY 493/503 and TG contents in the ZFL cells. **(C)** Immunoblot and densitometric analysis of LC3 and p62 in the ZFL cells. (^∗^*P* < 0.05, *n* = 6). Error bars, SEM. **(D)** Co-localization of BODIPY 493/503 (green) with LC3 (red) in treated samples with CQ.

**FIGURE 4:**
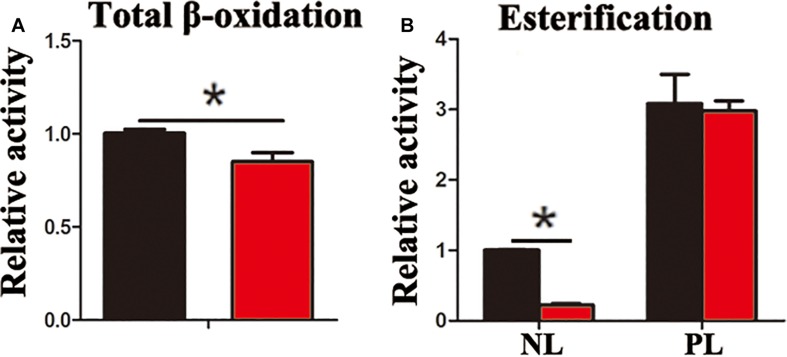
Inhibited lipophagy reduces activities of lipid catabolism and esterification in ZFL cell line. **(A)** The β-oxidation activities of [1-^14^C] oleic acid in the ZFL cells. **(B)** The esterification activities of [1-^14^C] oleic acid in the ZFL cells. (^∗^*P* < 0.05, *n* = 4). Error bars, SEM.

In the present study, the accumulation of LDs and cellular TG content increased significantly after pharmacological inhibition of autophagy by using CQ in ZFL cells (Figures 3A,B). Moreover, inhibiting autophagy by using CQ also increased the markers of blocked autophagy such as LC3-II and p62 protein levels (Pankiv et al., 2007; Larsen et al., 2010), showing autophagy was inhibited in ZFL cells (Figure 3C). Since CQ mainly inhibits degradation of LC3-II, it was reasonable for LC3-II protein to accumulate more in inhibited ZFL cells. Double immunofluorescence studies also indicated significant co-localization of LDs with LC3 in CQ-treated cells than in non-CQ-treated cells, suggesting that CQ-induced dysfunction of lysosomes caused accumulation of LC3 and LDs by suppressing lipophagy (Figure 3D). We further conducted biochemical assays, which indicated clearly that the CQ-caused lipophagy inhibition decreased significantly the FA β-oxidation efficiency (Figure 4A) and the FA esterification (Figure 4B) into neutral lipids (NL) in ZFL cells. It is known that, lipophagy is an important cellular process, which breaks down TG and releases FFAs (Singh et al., 2009; Skop et al., 2012). The FFAs released are used as common substrates for either FA β-oxidation or esterification. Therefore, the inhibited lipophagy reduces the supply of FFAs for lipid metabolism and subsequently suppresses lipid metabolism. The data obtained from CQ inhibition reveal the important roles of lipophagy in lipid metabolism in fish liver cells, similar to results reported by Wang et al. (2018) in live zebrafish. More importantly, our present results and the previous study (Wang et al., 2018) both confirm that the dysfunction of lipophagy causes lipid accumulation and reduces lipid metabolism biochemical activities in cells, either in the fed or fasted states, indicating that, lipophagy plays various roles in different nutritional states. In order to investigate thoroughly the functions of autophagy in nutrients metabolism, genetically modified animals, or cell models are required in future studies, because our present study and previous report (Wang et al., 2018) both inhibited autophagy by using pharmacological inhibitors.

### Inhibited Lipophagy Suppressed the Expression of Lipid Metabolism-Related Genes in ZFL Cells

**FIGURE 5:**
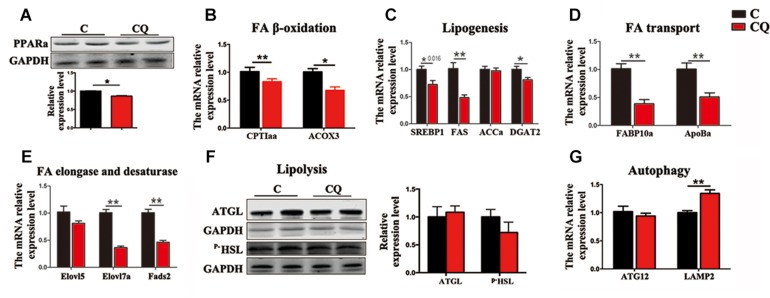
Inhibited lipophagy suppresses expression of lipid metabolism-related genes and protein levels in ZFL cell line. **(B–E,G)** The mRNA expression of the genes related to lipid metabolism. **(A,F)** The protein levels of PPARa, ATGL and p-HSL. ^∗^*P* < 0.05, ^∗∗^*P* < 0.01, *n* = 6. Error bars, SEM.

The expression of the genes and proteins involved in FA β-oxidation further showed that CQ treatment decreased considerably PPARα protein (Figure 5A) and the mRNA expression of carnitine palmitoyltransferase 1aa (*CPT1aa*) and acyl-coenzyme A oxidase 3 (*ACOX3*) in ZFL cells (Figure 5B). We reasoned that the decrease in TG-sourced FFAs after autophagy inhibition resulted in the downregulation of FFAs breakdown-related genes and β-oxidation activity. This is because endogenous FFAs are ligands for a series of nucleus receptors, such as PPARα, which triggers FA breakdown through β-oxidation (Schopfer et al., 2010; Fiamoncini et al., 2015). Moreover, inhibited autophagy also decreased the mRNA expression levels of the genes involved in lipogenesis [sterol-regulatory element binding protein 1 (*SREBP1*)], fatty acid synthase (*FAS*) and diacylglycerol acyltransferase-2 (*DGAT2*)] (Figure 5C), FA transport [fatty acid binding protein 10a (*FABP10a*) and apolipoprotein Ba (*ApoBa*)] (Figure 5D), FA elongation and desaturation [fatty acid elongase 7a (*Elovl7a*) and fatty acid desaturase 2 (*Fads2*)] (Figure 5E). These results indicate that, inhibited lipophagy downregulated most biochemical pathways in lipid metabolism. It is worth noting that, inhibited lipophagy did not significantly affect the lipolysis-related protein levels such as ATGL and ^*p–*^HSL (Figure 5F). The unchanged lipolysis protein levels suggest that, the reduced FFAs supply due to inhibited lipophagy can not be compensated by increased lipolysis in ZFL cells because lipolysis is an important biochemical reaction required for the release of FFAs other than lipophagy.

We further noticed that CQ treatment also increased the mRNA expression of lysosomal associated membrane protein 2 (*LAMP2*) gene (Figure 5G), a membrane receptor involved in chaperone-mediated autophagy (Glick et al., 2010), reflecting an increased compensatory autophagic pathway against macroautophagy (lipophagy). However, inhibited lipophagy is the main reason for the lowered lipid metabolism in ZFL cells because chaperone-mediated autophagy mainly plays roles in protein degradation (Majeski and Fred Dice, 2004), but not in TG breakdown.

## Conclusion

Taken together, our present study verified that lipophagy exists in ZFL cells and it can be activated by starvation as in live fish. The dysfunction of lipophagy decreased substantially the lipid catabolism and anabolism in fish liver cells at both fed and starved states, indicating that lipophagy is an important cellular process in lipid metabolism in fish livers. Therefore, lipophagy might be a regulatory target in fatty liver prevention or treatment in farmed fish.

## Author Contributions

Z-YD, JW, D-LLi, and M-LZ conceived the study. JW, S-LH, L-YL, and D-LLu performed the experiments. JW analyzed the data. JW, SL, and Z-YD wrote the manuscript. All authors approved the final version of the manuscript.

## Conflict of Interest Statement

The authors declare that the research was conducted in the absence of any commercial or financial relationships that could be construed as a potential conflict of interest.
