# Supplementary material for: Inhibited Lipophagy Suppresses Lipid Metabolism in Zebrafish Liver Cells
Source: Front Physiol. 2019 Aug 21;10:1077. doi: 10.3389/fphys.2019.01077 (PMC6713122; doi:10.3389/fphys.2019.01077)
Supplement: Supplementary file 1 [file Table_1.docx]

**Table**

**Table S1. Sequences of the primer pairs for qRT-PCR in ZFL cell line**

| Gene name | Primers (5’-3’) | GenBank NO. |
| --- | --- | --- |
| Elongation factor 1 α (EF1α) | F:CCCCTGGACACAGAGACTTCATC | NM_131263.1 |
|  | R:ATACCAGCCTCAAACTCACCGAC |  |
| β-actin | F:TCTGGTGATGGTGTGACCCA | NM_131031.2 |
|  | R:GGTGAAGCTGTAGCCACGCT |  |
| Microtubule-associated protein 1 light chain 3b (LC3b) | F:CAGCGGGTGGAGGATGTA | NM_199604.1 |
|  | R:AACGTGGTCAGGAACTAGAAAC |  |
| Autophagy related 12 (ATG12) | F:GCCATCTCACGCTTCCTCA | NM_001246200.1 |
|  | R:GCCGTCACTTCCGAAACACT |  |
| Lysosomal-associated membrane protein 2 (LAMP2) | F:CTCCTCCGACGACTCCTTCT | XM_009291116.3 |
|  | R:GGCTGGCGTTCCCACTTA |  |
| FFA binding protein 10a (FABP10a) | F:GGCTGCAGAACTGACCGAT | NM_152960.1 |
|  | R:TGATCATGGTGGTTCCTCCG |  |
| Apolipoprotein Ba (ApoBa) | F:TGAGAATGGGGCTTTGGGTC | XM_689735.9 |
|  | R:TATGTCCTGAGGGACGGGAA |  |
| Fatty acid desaturase 2 (Fads2) | F:CAGCATCACGCTAAACCCAAC | NM_131645.2 |
|  | R:AGGGGAGGACCAATGAAGAAG |  |
| Fatty acid elongase 5 (Elovl5) | F:TGTCTGACGCTGCTGTCTCTCTAT | NM_200453.2 |
|  | R:ATCGGCGTCTCCTCCACTGT |  |
| Fatty acid elongase 7a (Elovl7a) | F:TGAAGCGGGTCCTCATCGTT | NM_199875.1 |
|  | R:TCCCCAGCCTGCCATCAA |  |
| Sterol regulatory element binding transcription factor 1 (SREBP1) | F:CAGAGGGTGGGCATGCTGGC | NM_001105129.1 |
|  | R:ATGTGACGGTGGTGCCGCTG |  |
| Fatty acid synthase (FAS) | F: GGAGCAGGCTGCCTCTGTGC | XM_009306806.3 |
|  | R: TTGCGGCCTGTCCCACTCCT |  |
| Acetyl-CoA carboxylase α (ACCα) | F: GCGTGGCCGAACAATGGCAG | XM_021476200.1 |
|  | R: GCAGGTCCAGCTTCCCTGCG |  |
| Diacylglycerol O-acyltransferase 2 (DGAT2) | F: ACGCATAACCTGCTTCCC | NM_001030196.1 |
|  | R: TCCTGTGGCTTCTGTCCC |  |
| Carnitine palmitoyltransferase 1aa (CPT1aa) | F: CATCCTTAGGCCTGCTCTTCAAA | NM_001044854.1 |
|  | R:ACCATGACACCCCCAACTAACAT |  |
| Acyl-CoA oxidase 3 (ACOX3) | F: TGGAAGGACATGATGCGCTTT | NM_213147.1 |
|  | R: AGGCTGCCGGGCAAAAA |  |
